# Ag-Incorporated Cr-Doped BaTiO_3_ Aerogel toward Enhanced Photocatalytic Degradation of Methyl Orange

**DOI:** 10.3390/nano14100848

**Published:** 2024-05-13

**Authors:** Jun Wu, Gaofeng Shao, Xiaodong Wu, Sheng Cui, Xiaodong Shen

**Affiliations:** 1 College of Materials Science and Engineering, Nanjing Tech University, Nanjing 211816, China; wujun890329@163.com (J.W.); wuxiaodong@njtech.edu.cn (X.W.); cui2002sheng@126.com (S.C.); 2 Jiangsu Collaborative Innovation Center for Advanced Inorganic Function Composites, Nanjing Tech University, Nanjing 211816, China; 3 School of Chemistry and Materials Science, Nanjing University of Information Science & Technology, Nanjing 210044, China

**Keywords:** BaTiO_3_ aerogel, Cr-doped, Ag nanoparticles, photocatalytic degradation, methyl orange removal

## Abstract

A novel Cr-doped BaTiO_3_ aerogel was successfully synthesized using a co-gelation technique that involves two metallic alkoxides and a supercritical drying method. This freshly prepared aerogel has a high specific surface area of over 100 m^2^/g and exhibits improved responsiveness to the simulated sunlight spectrum. Methyl orange (MO) was chosen as the simulated pollutant, and the results reveal that the Cr-doped BaTiO_3_ aerogel, when modified with the noble metal silver (Ag), achieves a pollutant removal rate approximately 3.2 times higher than that of the commercially available P25, reaching up to 92% within 60 min. The excellent photocatalytic performance of the Ag-modified Cr-doped BaTiO_3_ aerogel can be primarily attributed to its extensive specific surface area and three-dimensional porous architecture. Furthermore, the incorporation of Ag nanoparticles effectively suppresses the recombination of photo-generated electrons and holes. Stability and reusability tests have confirmed the reliability of the Ag-modified Cr-doped BaTiO_3_ aerogel. Therefore, this material emerges as a highly promising candidate for the treatment of textile wastewater.

## 1. Introduction

Water contamination is a prominent and pressing issue resulting from industrial activities [1,2,3]. The textile industry, in particular, significantly contributes to global wastewater by releasing different types of organic dyes into water bodies like rivers, lakes, and oceans. These dyes persist in the environment due to their resistance to degradation caused by light, temperature, and various environmental conditions, thereby posing long-term ecological risks. As this problem continues to worsen, obtaining clean water for basic human needs becomes increasingly challenging [4]. However, the photocatalytic degradation of organic contaminants provides a promising solution for environmental cleanup. This method offers several advantages, including cost-effectiveness, absence of secondary pollution, and straightforward processing techniques [5,6].

Through a series of oxidation reactions, the colored organic dyes found in industrial wastewater can be completely degraded into water, carbon dioxide, or other harmless colorless organic compounds. This sets them apart from traditional binary oxide semiconductors, as ternary or multicomponent semiconductors offer a greater number of modification sites, resulting in superior functional adaptability. Consequently, these semiconductors are becoming increasingly important in the field of photocatalytic materials research [5]. One standout among the various photocatalytic materials is barium titanate (BaTiO_3_), which serves as an effective semiconductor photocatalyst [7]. Its high efficiency stems from its perovskite structure, optimal alignment of the valence band (VB) and conduction band (CB), availability in various sizes and shapes, environmental friendliness, and robust stability. Nevertheless, conventional BaTiO_3_ photocatalysts suffer from several limitations, including low specific surface area, limited adsorption capacity, and the need for high heat treatment temperatures [8,9]. However, by combining BaTiO_3_ with aerogel, these drawbacks can be overcome, greatly expanding its potential in photocatalytic applications.

As a nanoporous material with a continuous three-dimensional network structure, aerogel has shown great potential in adsorption and photocatalysis due to its high specific surface area and abundant pore structure [10,11,12]. In recent years, we have witnessed extensive research on the photocatalytic properties of aerogels. TiO_2_ [13], TiO_2_-SiO_2_ [14], CdS [15], SnO_2_ [16], ZnO [17], and other aerogels have been synthesized, and their photocatalytic degradation properties on dyes have been investigated. Significantly improved photocatalytic performance has been observed in aerogel systems compared to their corresponding nanocrystalline powders. For instance, TiO_2_ aerogel exhibits 1.6 times higher photocatalytic degradation performance for methyl orange (MO) compared to P25 nanoparticles [18]. CdS aerogel, on the other hand, has a specific surface area that is 3.4 times higher than that of CdS nanocrystals and shows 2.8 times higher degradation performance for Methylene blue (MB) [15]. Although there has been extensive research on the production of aerogels using binary oxide semiconductors for photocatalysis, studies on ternary oxide semiconductors have mainly focused on the use of high surface area aerogels, such as graphene aerogels, to incorporate photocatalytically active components [19]. Liu et al. [20] have successfully fabricated three-dimensional (3D) CeVO_4_/rGO porous aerogels through a one-pot hydrothermal method. The photocatalytic activities of these aerogels were investigated by studying the degradation of MB under visible light irradiation. Compared to bare CeVO_4_ particles, the CeVO_4_/rGO aerogels showed significantly improved photocatalytic efficiency, which could primarily be attributed to their larger surface area and porous structure. In addition, Liu et al. [21] synthesized Bi_2_MoO_6_/reduced graphene oxide aerogel (BMO/GA) composites that exhibited enhanced adsorptivity and photocatalysis, with a removal rate for MB that was about 2.1 times higher than that of pure BMO. However, the catalytic efficiency of these methods is limited by the ability of the graphene aerogel matrix to support a sufficient amount of photocatalytically active components. A more promising approach, albeit with several obstacles to overcome, would be the direct incorporation of photocatalytically active ingredients into aerogels.

The conventional synthesis process for aerogels involves five steps: sol–gel, solution displacement, aging, supercritical drying, and heat treatment [10]. This proposed approach can be applied to binary oxide aerogels like SiO_2_, TiO_2_, Al_2_O_3_, etc., which maintain a continuous three-dimensional network structure even after undergoing high-temperature calcination [22,23,24]. However, synthesizing ternary oxide aerogels like BaTiO_3_ using this approach is challenging. The amorphous gels that are initially prepared require a significant increase in temperature to fully crystallize, at which point the skeleton structure of the material collapses, making it difficult to form an aerogel [25]. To overcome material shrinkage, it is typically necessary to use templates or enhanced synthesis techniques. Wang et al. [26] created an InVO_4_ aerogel using the PMMA template method and utilized it for the photocatalytic degradation of methylene blue. Despite the improved catalytic efficiency compared to bulk InVO_4_, the specific surface area of the sample after calcination at 500 °C was limited to 35–52 m^2^/g, restricting further enhancements in catalytic efficiency. Chau et al. [27] used chitin nanocrystals as a liquid crystal template to synthesize BaTiO_3_ aerogel. However, removing the chitin template required high-temperature treatment at 900 °C, resulting in significant sample shrinkage and a reduced specific surface area of only 50 m^2^/g. The photocatalytic degradation efficiency towards methylene blue showed a slight improvement compared to that of P25. Li et al. [28] produced a BaTiO_3_ aerogel with a high surface area of 233 m^2^/g using an assembly method, and evaluated its photocatalytic efficiency in the degradation of MO under ultraviolet irradiation. However, the preparation process was relatively complex. Both of these BaTiO_3_ aerogels also exhibited a limited response to the ultraviolet spectrum (approximately 3–5% of sunlight), had a wide band gap of 3.2 eV, and demonstrated high rates of recombination of photoinduced charged species (electrons and holes) [29]. Demydov et al. [30] employed a mixture of two metal alkoxides to improve gelation and successfully synthesized SrTiO_3_ and BaTiO_3_ aerogels with specific surface areas of 159 m^2^/g and 175 m^2^/g, respectively. The experimental procedure was straightforward. However, no investigation of the photocatalytic degradation performance was conducted.

In this study, we have successfully synthesized BaTiO_3_ aerogels by co-gelling two metallic alkoxides and utilizing the supercritical drying method. The preparation process of aerogel solely entails facile agitation and supercritical drying, rendering it highly amenable to industrialization. The as-prepared aerogel photocatalyst through a template-free synthetic route exhibits an exceptional surface area and abundant pore structure. On the one hand, we intentionally introduced impurity energy levels by incorporating Cr(NO_3_)_3_ during the gelation process to enhance the spectral response range of the aerogel. On the other hand, the deposition of noble metal nanoparticles (Ag) on the surface of Cr-doped BaTiO_3_ aerogels facilitated electron trapping, thereby ensuring an elevated level of photocatalytic efficiency. The resulting samples demonstrated outstanding photocatalytic efficiency in the degradation of MO under visible light irradiation.

## 2. Materials and Methods

### 2.1. Materials

Titanium (IV) isopropoxide (95%), deionized water, Cr(NO_3_)_3_·9H_2_O (99.95%), barium metal (99%), and MO (96%) were purchased from Aladdin Industrial Inc. (Shanghai, China). Absolute ethanol (99.7%) was supplied by Wuxi Yasheng Chemical Co., Ltd. (Wuxi, China). Toluene (99.7%) was purchased from Shanghai Lingfeng Chemical Reagent Co., Ltd. (Shanghai, China). AgNO_3_ (99.8%) was purchased from Sinopharm Chemical Reagent Co., Ltd. (Shanghai, China). All chemicals were of analytical reagent grade and used as received without any additional purification.

### 2.2. Synthesis of BaTiO_3_ Aerogel

Prior to conducting the experiment, it is necessary to remove the oxide film from the barium metal. To generate a metal alkoxide solution, 6 mmol of barium metal was initially dissolved in 25 mL of absolute ethanol. This solution was then mixed with 6 mmol of titanium isopropoxide and 35 mL of toluene, resulting in a clear solution. The mixture was subsequently stirred in a sealed container for 0.5 h. Hydrolysis was induced by adding 36 mmol of deionized water, and the resulting solution was left at room temperature with continuous stirring for at least 12 h. Over this time, the solution transformed into a slightly milky wet gel. Lastly, the gel mixture was transferred into four 20 mL glass bottles and dried under supercritical C_2_H_5_OH conditions (10 MPa, 270 °C) for 2 h, resulting in the production of BaTiO_3_ aerogels.

### 2.3. Synthesis of Cr-Doped BaTiO_3_ Aerogel

The synthesis procedure for the Cr-doped BaTiO_3_ aerogel is similar to that used for the BaTiO_3_ aerogel. To obtain a stoichiometric ratio of Ba_0.99_Cr_0.01_TiO_3_, 5.94 mmol of barium metal was dissolved in 25 mL of absolute ethanol to form a metal alkoxide solution. Then, 6 mmol of titanium isopropoxide and 35 mL of toluene were added to this solution to create a clear solution. The mixture was stirred in a sealed container for from 0.5 to 1 h, during which 0.06 mmol of Cr(NO_3_)_3_·9H_2_O was dissolved. Hydrolysis was then induced by adding 36 mmol of deionized water. The resulting solution was left to stir at room temperature for at least 12 h until it transformed into a light green wet gel. Subsequently, the mixture was transferred into four 20 mL glass bottles and dried under supercritical C_2_H_5_OH (10 MPa, 270 °C) conditions for 2 h to produce Cr-doped BaTiO_3_ aerogels. By adjusting the molar ratio of Ba and Cr, it is possible to synthesize Ba_0.95_Cr_0.05_TiO_3_ and Ba_0.9_Cr_0.1_TiO_3_ using the same method. The three samples were named BTO-Cr001, BTO-Cr005, and BTO-Cr010 after preparation.

### 2.4. Deposition of Noble Metal Ag Nanoparticles on Aerogel Surface

Ag nanoparticles were synthesized using the photochemical deposition method and deposited onto the surface of BTO-Cr010 aerogel. A 50 mL solution of 0.001 M AgNO_3_ was prepared, and 100 mg of the aerogel sample was added to the solution. The mixture was stirred continuously under a 500 W ultraviolet lamp for 1 h to facilitate photochemical deposition. To remove any residual solution, the deposited sample was washed three times with deionized water using centrifugation at 5000 rpm. Afterward, the sample was immersed in an ethanol solution, resulting in a weight percentage of approximately 5 wt% of silver deposition, and was designated as 5% Ag/BTO-Cr010.

### 2.5. Photocatalytic Experiment

The photocatalytic activity of the synthetic chromium-doped BaTiO_3_ aerogels was assessed by measuring the photodegradation of a solution of methylene orange (MO) under simulated solar light at room temperature. In a typical photocatalytic experiment, we dispersed 100 mg of the Cr-doped BaTiO_3_ aerogel in 100 mL of a 10 mg/L MO solution. To adjust the pH to 3, we used 1 M HCl. Prior to illumination, the mixture was magnetically stirred in the dark at 150 rpm for 0.5 h to establish an adsorption–desorption equilibrium between the MO and the aerogel. For the light source, we utilized a solar simulator equipped with a 420 W xenon lamp. The mixed solution was positioned 300 mm away from the light source within a glass container. To analyze the reaction progress, we extracted a 5 mL suspension every 10 min. We then centrifuged the suspension at a speed of 5000 rpm for 10 min and collected the resulting clear solution. We determined the concentration of the collected clear solution by measuring the absorbance of MO at 463 nm using a 722S UV-visible spectrophotometer (Jinghua, Shanghai, China).

### 2.6. Characterization Methods

The X-ray diffraction (XRD) patterns were collected using a Rigaku Smart Lab 3000 diffractometer (Rigaku, Tokyo, Japan) with Cu Kα radiation (λ = 0.15406 nm) and a scanning range of 10–70°. Scanning electron microscopy (SEM) was conducted using a LEO-1530VP field emission scanning electron microscope (LEO/Zeiss, Oberkochen, Germany) operated at 6–8 kV and a ZEISS Sigma 300 field-emission scanning electron microscope (Zeiss, Oberkochen, Germany) operated at 12 kV. The morphology of BaTiO_3_ aerogels was observed using a JEOL JEM-2100 transmission electron microscope (TEM, JEOL, Tokyo, Japan) operated at 100 kV. The Brunauer-Emmett-Teller (BET) specific surface areas, pore volume, and pore distribution were measured by nitrogen adsorption/desorption isotherms using a Micromeritics ASAP2020 surface area and pore distribution analyzer (Micromeritics, Norcross, GA, USA) after the samples were degassed in a vacuum at 120 °C for 8 h. The UV-vis diffuse reflectance spectra were measured using a UV-vis spectrophotometer (CARY 300, Agilent, Palo Alto, CA, USA) with BaSO_4_ as the background. X-ray photoelectron spectroscopy (XPS) (EscaLab 250Xi, Thermo Fisher, Waltham, MA, USA) was used to determine the surface chemical compositions and valence states of the aerogel samples. The binding energies were calibrated using containment carbon (C 1s = 284.8 eV).

## 3. Results

### 3.1. Synthesizing Process of Materials

**Figure 1:**
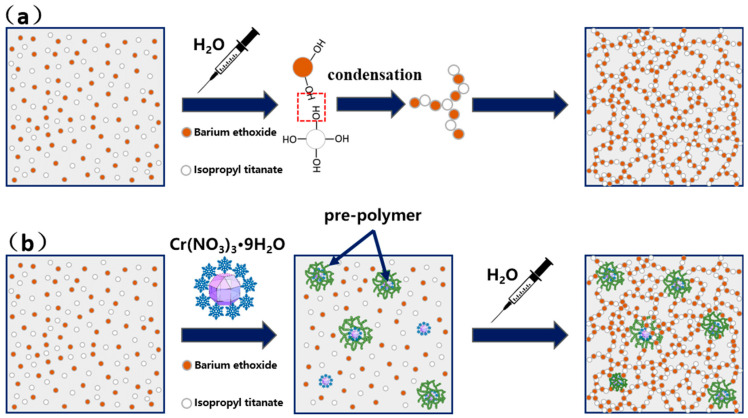
Schematic illustration of the synthesizing process of (**a**) pure BaTiO_3_ aerogel and (**b**) Cr-doped BaTiO_3_ aerogels.

Figure 1 shows the schematic illustration of the synthesizing process of the pure BaTiO_3_ and Cr-doped BaTiO_3_ aerogels. In the case of synthesizing a pure BaTiO_3_ aerogel, the hydrolysis rates of both metal alkoxides are essentially the same. Consequently, barium ethoxide and isopropyl titanate can hydrolyze to form Ba(OH)_2_ and Ti(OH)_4_ precursors within the mixture. When these precursors come into contact, they dehydrate and condense, resulting in the formation of a sol. The sol then continues to condense until it reaches a gel state, forming a three-dimensional network skeleton. The hydrolysis and polycondensation of barium ethylate and isopropyl titanate within the solvent proceed in a non-sequential manner, resulting in a gel framework with a random distribution (Figure 1a).

However, in the case of synthesizing a Cr-doped BaTiO_3_ aerogel, the water of crystallization serves as a hydrolytic agent for the pre-hydrolysis of barium ethylate and isopropyl titanate around chromium nitrate. Subsequently, Ba(OH)_2_ and Ti(OH)_4_ dehydrate and condense to form a series of pre-polymers. However, these pre-polymers are unable to crosslink with each other due to their low concentration, preventing the formation of a network skeleton. Only when deionized water is added as a hydrolytic agent can the unhydrolyzed metal alkoxides undergo a sol-gel process, similar to the process shown in Figure 1a, resulting in the formation of a three-dimensional network skeleton, as depicted in Figure 1b.

### 3.2. X-ray Diffraction

**Figure 2:**
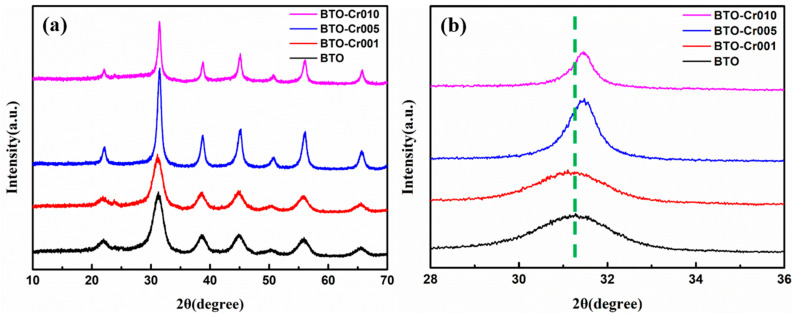
(**a**) XRD patterns of BTO, BTO-Cr001, BTO-Cr005, and BTO-Cr010. (**b**) Diffraction peak shift for the (110) plane.

The X-ray diffraction (XRD) patterns for the pure BaTiO_3_ aerogel and Cr-doped BaTiO_3_ aerogels are illustrated in Figure 2a. The results indicate that the diffraction peaks of the pure BaTiO_3_ aerogel, positioned at 21.9° (100), 31.3° (110), 38.7° (111), 45.0° (200), 50.7° (210), 55.9° (211), and 65.5° (220), correspond well to the cubic phase of BaTiO_3_ (JCPDF 31-0174). In the XRD patterns of the Cr-doped BaTiO_3_ aerogels, the corresponding diffraction planes of the BaTiO_3_ phase exhibit a peak shift to the right, as shown in Figure 2b. This shift can be attributed to the introduction of chromium into the crystal lattice. The smaller size of chromium in comparison to barium creates stress or strain in the crystal lattice [31]. The cell parameters and crystalline sizes of Cr-doped BaTiO_3_ aerogels were calculated by the MDI Jade 6.0 package. The cell parameters are 4.03 Å (BTO), 4.02 Å (BTO-Cr001), 4.01 Å (BTO-Cr005), and 4.01 Å (BTO-Cr010). This suggests that the incorporation of chromium does not significantly alter the BaTiO_3_ structure, but rather causes a lattice shrinkage through the substitutional incorporation of chromium. The crystalline sizes are 6.2 nm (BTO), 5.0 nm (BTO-Cr001), 12.8 nm (BTO-Cr005), and 10.6 nm (BTO-Cr010). As discussed in Figure 1b, as the Cr content increases, the pre-polymers grow more fully, thereby promoting the formation of larger crystal grains. Therefore, the diffraction peaks at approximately 31° for the bare and 0.01 doped samples are broadened and the others are sharp.

### 3.3. XPS Analysis

**Figure 3:**
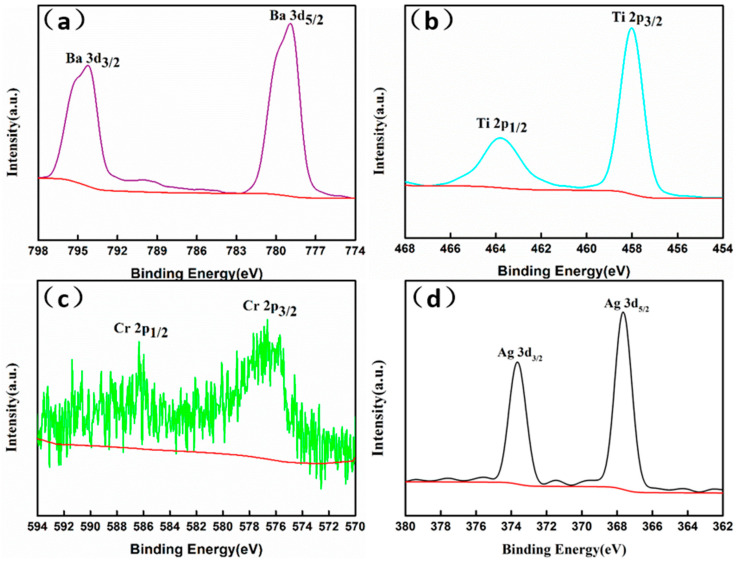
XPS spectra of Ag-incorporated Cr-doped BaTiO_3_ aerogel. (**a**) Ba 3d, (**b**) Ti 2p, (**c**) Cr 2p, and (**d**) Ag 3d.

XPS is an effective technique for determining the surface chemical compositions and valence states of a material. Figure 3 presents the high-resolution XPS spectrum of Ba 3d, Ti 2p, Cr 2p, and Ag 3d for an Ag-incorporated Cr-doped BaTiO_3_ aerogel. In the high-resolution XPS spectrum of the Ba 3d region (Figure 3a), the characteristic peaks at 793.9 eV and 778.6 eV can be attributed to the Ba 3d_3/2_ and Ba 3d_5/2_ electron levels, respectively, confirming the oxidation state of Ba^2+^ in the BTO-Cr010 aerogel. Figure 3b displays the Ti 2p_1/2_ and Ti 2p_3/2_ electron levels, exhibiting binding energies at 464.4 eV and 458.8 eV, which are consistent with those previously reported for Ti^4+^ in BaTiO_3_ materials [32]. The typical Cr 2p spectrum in Figure 3c indicates the presence of Cr 2p_1/2_ and Cr 2p_3/2_ in the Cr^3+^ chemical states, corresponding to their binding energies at 586.3 eV and 576.5 eV, respectively [33]. The absence of the characteristic peak at 579.3 eV in the Cr 2p spectrum implies the non-existence of Cr^6+^ within the BTO-Cr010 aerogel, ensuring that the Cr-doped BaTiO_3_ aerogel is environmentally friendly. It is worth noting that, while Cr^3+^ is considered an essential trace element for human physiological functions, Cr^6+^ is one of the toxic metal ions commonly found in the environment [34]. As shown in Figure 3d, the Ag structure consists of two peaks at the surface, which are located at 373.83 and 367.83 eV accounting for Ag 3d3/2 and Ag 3d5/2, respectively. The spin–orbit splitting energy of the 3d doublet is 6.0 eV. The disparity in binding energies suggests that silver predominantly exists in the Ag0 state [35]. The atomic compositions, as determined by XPS analysis, are as follows: Ba (16.12%), Cr (1.72%), Ti (16.61%), O (63.15%), and Ag (2.39%).

### 3.4. Morphology and Microstructure

**Figure 4:**
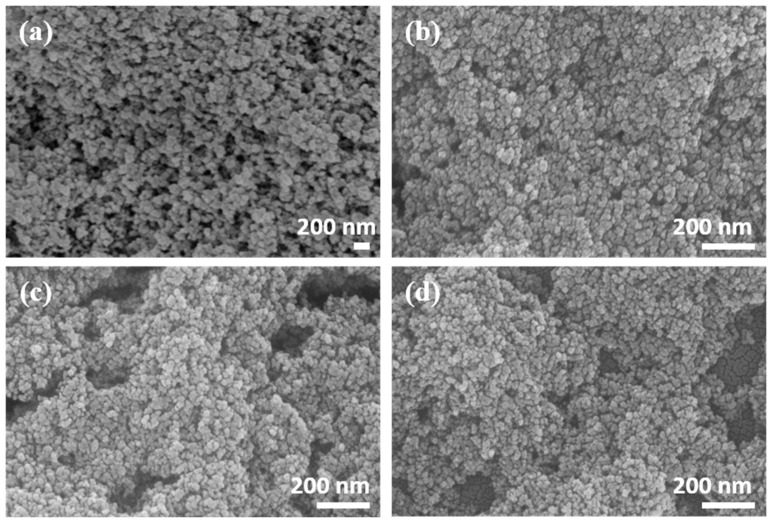
SEM micrographs of (**a**) BTO, (**b**) BTO-Cr001, (**c**) BTO-Cr005, and (**d**) BTO-Cr010.

The morphology of the pure BaTiO_3_ aerogel and Cr-doped BaTiO_3_ aerogels was analyzed using a field-emission scanning electron microscope. Figure 4a illustrates the typical continuous three-dimensional network structure of the aerogel, confirming the successful synthesis of a pure BaTiO_3_ aerogel through the co-gelling of two metallic alkoxides and the supercritical drying method. The introduction of chromium led to an increase in the crosslinking degree between the skeleton, resulting in a slight deterioration of the pore structure distribution (Figure 4b–d).

**Figure 5:**
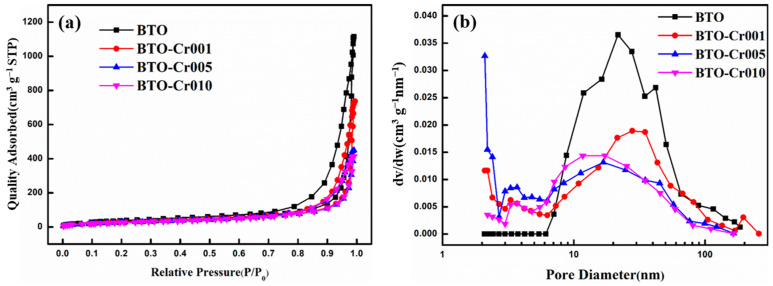
(**a**) Nitrogen adsorption/desorption isotherms and (**b**) pore-size distribution curves of pure BaTiO_3_ aerogel and Cr-doped BaTiO_3_ aerogels.

The surface area and porosity characteristics of the pure BaTiO_3_ aerogel and Cr-doped BaTiO_3_ aerogels were determined through N_2_ adsorption/desorption experiments. Figure 5 displays the nitrogen adsorption/desorption isotherms and BJH pore-size distributions. All the aerogels exhibited type-IV isotherms, which is typical for mesoporous materials. The observed hysteresis loop with an H-1 shape indicates the presence of capillary condensation associated with mesopores, confirming the successful synthesis of a three-dimensional cross-linked structure. The specific surface area was measured using the BET method, and the BET-specific surface areas of BTO, BTO-Cr001, BTO-Cr005, and BTO-Cr010 were calculated to be 120.7, 109.8, 108.8, and 107.2 m^2^/g, respectively. These values were significantly higher than those of the BaTiO_3_ aerogels obtained via the template method [27]. The increased specific surface area provides a larger number of surface-active sites, facilitating the migration of charge carriers and thereby enhancing photocatalytic performance. Additionally, the pore-size distribution was calculated using the classical Barrett–Joyner–Halenda (BJH) model, as shown in Figure 5b. The mesopore diameters of BTO, BTO-Cr001, BTO-Cr005, and BTO-Cr010 were estimated to be 30.4, 29.8, 20.4, and 21.0 nm, respectively. It is evident that the mesoporous diameter of the aerogel decreases after Cr doping, which aligns with the observations from scanning electron microscope (SEM) micrographs. Furthermore, the nanoporous structure of both the pure BaTiO_3_ aerogel and the Cr-doped BaTiO_3_ aerogels facilitates the adsorption of reactants and transportation of products, thereby further improving the photocatalytic performance.

**Figure 6:**
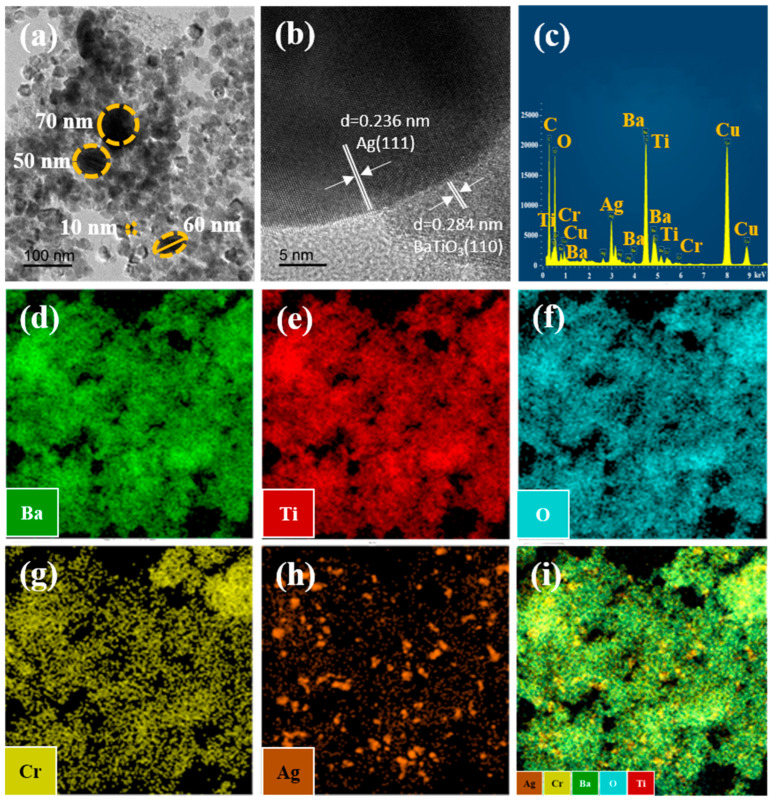
(**a**) TEM micrographs, (**b**) HRTEM micrographs, (**c**) STEM-EDX images, and (**d**–**i**) mapping images of 5% Ag/BTO-Cr010.

The TEM micrographs and mapping images of the 5% Ag/BTO-Cr010 are presented in Figure 6. As shown in Figure 6a, the BaTiO_3_ particles have a uniform grain size of approximately 10 nm, while the Ag nanoparticles exhibit varying particle sizes ranging from 10 to 70 nm. Further crystallographic features were identified using HR-TEM. Fourier transforms of the high-resolution image were used to measure inter-reticular distances. Figure 6b displays the lattice image of a particle with a D-spacing of 0.284 nm and 0.236 nm, corresponding to the cubic BaTiO_3_ structure (110 plane) and elemental silver (111 plane), respectively. This indicates that the deposited Ag exists in its elemental form as Ag^0^ [35], in agreement with the XPS results. The STEM-EDX image of the aerogel is shown in Figure 6c, where the peaks of copper belong to the target stand, and the peaks of carbon belong to the carbon adhesive. The atomic percentages of all elements (excluding copper and carbon) are as follows: Ba (14.15%), Cr (1.43%), Ti (16.17%), O (59.42%), and Ag (8.84%). The above results are highly consistent with the XPS analysis, except for a slightly higher atomic percentage of Ag. This bias may arise from the selection of specific regions for STEM characterization. Mapping images of the sample elements are shown in Figure 6d–i, depicting the spectrograms for Ba elements (Figure 6d), Ti elements (Figure 6e), O elements (Figure 6f), Cr elements (Figure 6g), and Ag elements (Figure 6h), as well as the overlapping spectrogram of all elements (Figure 6i). The distribution patterns of the Ba, Ti, and O elements exhibit remarkable similarity, indicating the presence of nanocrystals composed of BaTiO_3_. The uniform distribution of Cr elements in the nanocrystals of BaTiO_3_ suggests that the introduction of chromium during the preparation process of BaTiO_3_ aerogels enables homogeneous doping. Ag is observed to exist within the nanocrystals of BaTiO_3_ in varying particle sizes, ranging from a few nanometers to tens of nanometers, as depicted in Figure 6h.

### 3.5. Optical Characterization

**Figure 7:**
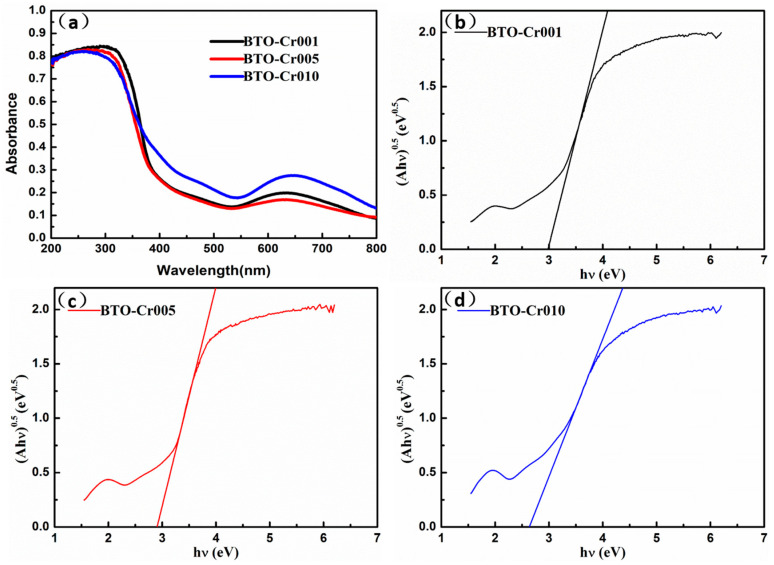
(**a**) UV–vis diffuse reflectance spectra (DRS) of Cr-doped BaTiO_3_ aerogels, (Ahν)^1/2^-hν curve for band gap calculation of (**b**) BTO-Cr001, (**c**) BTO-Cr005, and (**d**) BTO-Cr010.

The optical properties of Cr-doped BaTiO_3_ aerogels were assessed using UV-vis diffuse reflectance spectra (DRS), as shown in Figure 7. As expected, all Cr-doped BaTiO_3_ aerogels demonstrated photocatalytic activity across the UV to visible light spectrum. Figure 7a illustrates that the absorption edge exhibited a slight redshift with increasing amounts of Cr-doping, indicating an expanded light response range and enhanced utilization of solar energy. Figure 7b–d depicts the relationship curve (Ahν)^1/2^-hν, which was obtained using the formula proposed by Tauc, Davis, and Mott et al. Here, hν was set as the x-axis, (Ahν)^1/2^ was set as the y-axis, and the reverse extension of the tangent line intersected with the x-axis. The intersection on the x-axis represents the value of the optical bandgap [13]. Specifically, the calculated bandgaps for BTO-Cr001, BTO-Cr005, and BTO-Cr010 were 3.00 eV, 2.91 eV, and 2.72 eV, respectively. In comparison to pure BaTiO_3_ materials, which have a bandgap of 3.2 eV [36], Cr-doped BaTiO_3_ aerogels exhibit a reduced bandgap due to the incorporation of impurity levels. The former corresponds to electron transition from the valence band (VB) of BaTiO_3_ (O 2p orbital) to its conduction band (CB) (Ti 3d orbital) in a photoexcited state, while the latter is formed by electron transition from the impurity level (Cr 3d orbital) to the CB of BaTiO_3_ (Ti 3d orbital).

### 3.6. Photocatalytic Activity

**Figure 8:**
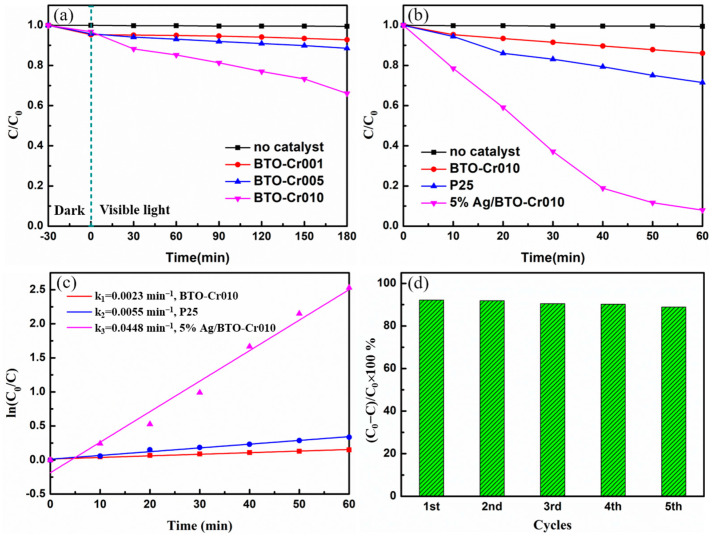
(**a**) Photodegradation curves of no catalyst and Cr-doped BaTiO_3_ aerogels toward MO under visible light irradiation, (**b**) comparison of photodegradation efficiency of BTO-Cr010, P25, and 5% Ag/BTO-Cr010 (without adsorption) toward MO under visible light irradiation, (**c**) plots of ln(C_0_/C) vs. irradiation time (t) for the BTO-Cr010, P25, and 5% Ag/BTO-Cr010. (**d**) The cycling degradation efficiency of 5% Ag/BTO-Cr010 toward MO under visible-light irradiation.

The photocatalytic activity of the Cr-doped BaTiO_3_ aerogels was evaluated by studying the photodegradation of MO under simulated sunlight irradiation using a 420 W xenon lamp with a filter, which can only allow light with a wavelength range of 400–780 nm to pass through. The results, shown in Figure 8a, indicate that MO is a photostable component, as no change was observed after solely being exposed to visible light for 180 min. BTO-Cr001 and BTO-Cr005 exhibit weak visible light photodegradation performance, with a degradation rate of MO within 3 h of less than 10%. This limitation can be attributed to their wide bandgaps of 3.00 eV and 2.91 eV, respectively, which only allow for a limited response to the simulated sunlight spectrum (approximately 3–7%). On the contrary, BTO-Cr010, with a narrow bandgap of 2.72 eV, exhibits a wider spectral response range and significantly improved photodegradation efficiency. The degradation rate of MO within 3 h can reach approximately 34%. However, compared to other catalysts reported in the literature, the visible light photocatalytic performance of BTO-Cr010 still falls short.

We propose that the primary limiting factor impeding the photocatalytic efficiency of Cr-doped BaTiO_3_ aerogels is the high recombination rates of photoinduced charged species (electrons and holes). Noble metal deposition can address this issue by enhancing the absorption of visible light through the surface plasmonic resonance absorption effect (SPR) and separating photogenerated charges via the formation of a Schottky barrier to facilitate electron trapping. This improves the photocatalytic performance of the sample [37]. Figure 8b illustrates the visible light photocatalytic degradation efficiency of the Cr-doped BaTiO_3_ aerogel with noble metal silver modification, as compared to the initial sample BTO-Cr010 and the commercial Degussa P25. The commercial Degussa P25 is included as a reference due to its distinctive heterojunction structure, which typically exhibits significant photocatalytic degradation capability under visible light. To closely align with practical application scenarios, the photocatalytic degradation experiment was initiated immediately after the dye and catalyst were mixed. The results show that the degradation rate of MO within 1 h is approximately 15% for the initial sample BTO-Cr010 and 30% for the commercial Degussa P25. Both the Cr-doped BaTiO_3_ aerogels without noble metal silver modification and the commercial Degussa P25 are inherently limited in visible light photodegradation. However, for the 5% Ag/BTO-Cr010 sample (with approximately 5 wt% silver deposition), MO can be almost completely degraded, with a degradation rate of 92%, which is 3.2-fold higher than that of commercial P25. This significant improvement expands the application potential of the material in the field of photocatalytic degradation.

To further investigate the photocatalytic activities of these samples, pseudo first-order kinetics were employed to analyze the photocatalytic degradation kinetics. The photodegradation reaction rate constant (k) was determined from the slope of the ln (C_0_/C) vs. time plot in Figure 8c. The linear behavior of the plot confirmed that the photodegradation reaction kinetics followed the first-order rate law. The photocatalytic degradation of MO catalyzed by the 5% Ag/BTO-Cr010 aerogel sample exhibited the highest reaction rate, with k = 0.0448 min^−1^, which was almost 19.5-fold higher than that of the initial BTO-Cr010 sample (k = 0.0023 min^−1^) and 8.1-fold higher than that of the commercial Degussa P25 (k = 0.0055 min^−1^). Table 1 lists the photocatalytic performance of various catalysts for the degradation of MO under visible light illumination. Using a bare BaTiO_3_ aerogel with a large specific surface area as the ground for photodeposited Ag nanoparticles has achieved impressive results. The 5% Ag/BTO-Cr010 aerogel exhibits superior or comparable photocatalytic degradation performance to other composite photocatalysts and commercialized P25.

The recycling and long-term stability of catalysts are crucial properties for practical applications. The stability and reusability of the Cr-doped BaTiO_3_ aerogel with noble metal silver modification were further assessed through additional experiments, as shown in Figure 8d. Under the same experimental conditions, after five cycles of photocatalytic degradation of MO, the 5% Ag/BTO-Cr010 aerogel still maintained 89% of the removal rate. These results demonstrate that the 5% Ag/BTO-Cr010 aerogel exhibits high cyclic stability for the photocatalytic degradation of MO.

### 3.7. Photocatalytic Mechanisms

**Figure 9:**
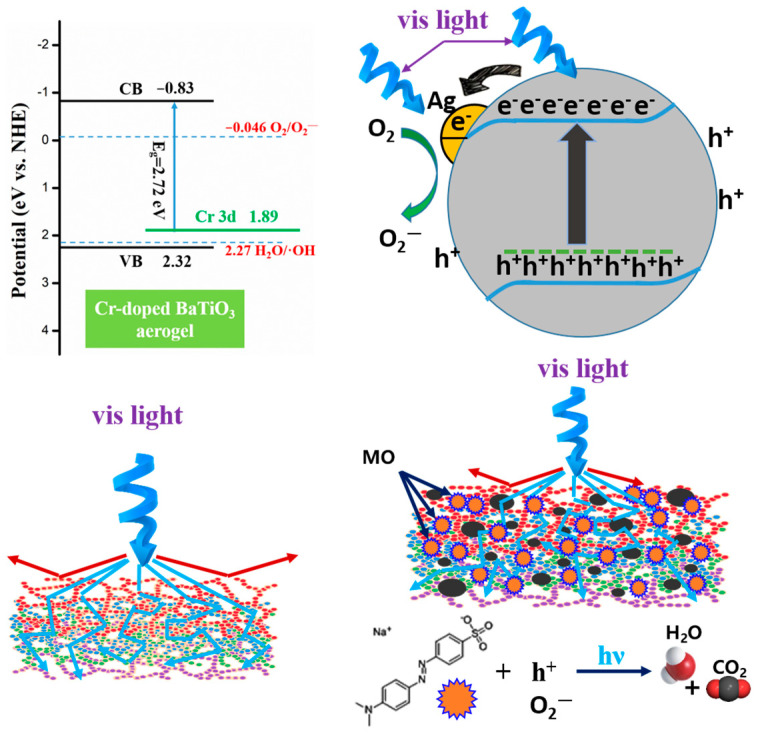
Schematic illustration of the band structure, charge separation, and photodegration process of 5% Ag/BTO-Cr010.

In order to comprehend the photocatalytic mechanism of Cr-doped BaTiO_3_ aerogels, it is imperative to determine their energy-band potentials, as the redox ability of photogenerated electrons and holes is closely associated with these potentials. The VB and CB potentials of BaTiO_3_ can be calculated by Equations (1) and (2):

(1) $$E_{VB}=\chi -E^{e}+0.5E_{g}$$

(2) $$E_{CB}=\chi -E^{e}-0.5E_{g}$$

where *E_VB_* and *E_CB_* are the VB edge and CB edge potentials (eV vs. NHE), and *χ* is the absolute electronegativity of the BaTiO_3_ aerogel. The term is defined as the arithmetic mean of the electron affinity and the first ionization energy of the constituent atoms and can be calculated to be 5.25 eV according to the literature [44]. *E^e^* is the energy of free electrons on the hydrogen scale (~4.5 eV). The bandgap energy *E_g_* of BaTiO_3_ aerogel is 3.15 eV. As a result, the VB edge and CB edge potentials are estimated to be 2.32 and −0.83 eV vs. normal hydrogen electrode (NHE), respectively. The calculated result for the impurity energy level (Cr 3d) is 1.89 eV. The results are shown in Figure 9. It is clearly visible that the *E_CB_* of the Cr-doped BaTiO_3_ aerogel is more negative than the standard redox potential of O_2_/$O_{2}^{-}$ (−0.046 eV vs. NHE) [45], resulting in the formation of a large amount of $O_{2}^{-}$ radicals.

The schematic diagram illustrating the photocatalytic mechanisms of the Cr-doped BaTiO_3_ aerogel with noble metal silver modification is presented in Figure 9. Upon visible light irradiation, photogenerated electrons and holes were produced by excitation of 5% Ag/BTO-Cr010. The photogenerated electrons were transferred from the impurity level Cr 3d orbital to the Ti 3d orbital (CB) and then migrated towards the metallic Ag, accumulating on its surface. These electrons could be rapidly transferred to the adsorbed oxygen on the Ag surface, leading to the generation of free oxygen radicals ($O_{2}^{-}$). Simultaneously, the photogenerated holes were transferred to the aerogel surface. Both h^+^ radicals and $O_{2}^{-}$ radicals have strong oxidizing abilities, enabling the complete oxidation of MO into H_2_O and CO_2_ [37]. Acting as photogenerated electron traps, Ag enhances the rate of electron transfer to molecular oxygen and inhibits the recombination of photogenerated electrons and holes [35]. Furthermore, the continuous porous three-dimensional network enhances the material’s light-capturing capability through multiple reflections [46]. As a result, these factors contribute to the improved photocatalytic activity of the Cr-doped BaTiO_3_ aerogel with noble metal silver modification.

## 4. Conclusions

The synthesis of a Cr-doped BaTiO_3_ aerogel involves the co-gelling of two metallic alkoxides and supercritical drying. All samples exhibit a specific surface area exceeding 100 m^2^/g, which facilitates improved reactant absorption and product transfer. The Cr-doped BaTiO_3_ aerogel demonstrates an enhanced response to a simulated sunlight spectrum and exhibits a visible light photodegradation ability for MO. Additionally, by incorporating an appropriate amount of Ag nanoparticles within the Cr-doped BaTiO_3_ aerogel, the composite photocatalysts demonstrate exceptional performance in the degradation of MO under visible light illumination. This performance is 3.2-fold higher than that of commercial P25. The improvement in photocatalytic activity is believed to be attributed to the Schottky barrier junction at the interface and the surface plasmon resonance effect of Ag. These findings suggest that the direct synthesis of photocatalytic active ingredients into aerogels, followed by noble metal modification, can significantly enhance the photocatalytic performance of BaTiO_3_-based nanocomposites. As a result, these materials show promise as candidates for textile wastewater treatment.

## Figures and Tables

**Table 1:** Photocatalytic performance of various catalysts for the degradation of MO under visible light illumination.

Photocatalyst	Morphology	Photocatalytic Performance	Photodegradation Kinetics	Ref
5% Ag/BTO-Cr010 (100 mg)	Aerogels	92% for MO, 60 min (10 mg/L, 100 mL)	0.0448 min^−1^	This work
BaTiF_6_ (—)	Fibrous network	98% for MO, 150 min (Not mentioned)	0.0169 min^−1^	[38]
Fe–Cr codoped BaTiO_3_ (50 mg)	Irregular NPs	94% for MO, 90 min (20 mg/L, 50 mL)	0.0303 min^−1^	[39]
Cu_2_O-Ag/AgBr (70 mg)	Octahedral NPs	~100% for MO, 90 min (10 mg/L, 70 mL)	0.0358 min^−1^	[40]
BaTiO_3_/rGO (50 mg)	Nanosheet and NPs	70% for MO, 20 min (0.05 mM, 50 mL)	0.0556 min^−1^	[41]
BaTiO_3_@g-C_3_N_4_ (0.5 g/L)	Irregular NPs	76% for MO, 360 min (5 mg/L)	Not mentioned	[42]
BaTiO_3_/In_2_S_3_ (0.5 g/L)	core–shell	93% for MO, 90 min (10 mg/L, 100 mL)	0.0334 min^−1^	[43]

## Data Availability

All data that support this work are included in this manuscript.
